# Prevalence and Methods for Assessment of Oropharyngeal Dysphagia in Older Adults: A Systematic Review and Meta-Analysis

**DOI:** 10.3390/jcm11092605

**Published:** 2022-05-06

**Authors:** Thanh-Nhan Doan, Wen-Chao Ho, Liang-Hui Wang, Fei-Chun Chang, Nguyen Thanh Nhu, Li-Wei Chou

**Affiliations:** 1 Department of Public Health, China Medical University, Taichung 406040, Taiwan; drchannhan@gmail.com (T.-N.D.); wcho@mail.cmu.edu.tw (W.-C.H.); 2 Department of Rehabilitation, Quang Nam Northern Mountainous Region General Hospital, Quang Nam 560000, Vietnam; 3 Department of Speech Language Pathology and Auditory, HungKuang University, Taichung 433304, Taiwan; wlhui0815@gmail.com; 4 Ph.D. Program for Aging, China Medical University, Taichung 404332, Taiwan; charmmyicy@hotmail.com; 5 Department of Physical Medicine and Rehabilitation, China Medical University Hospital, Taichung 404332, Taiwan; 6 Faculty of Medicine, Can Tho University of Medicine and Pharmacy, Can Tho 94117, Vietnam; ntnhu@ctump.edu.vn; 7 Department of Physical Therapy and Graduate Institute of Rehabilitation Science, China Medical University, Taichung 406040, Taiwan; 8 Department of Physical Medicine and Rehabilitation, Asia University Hospital, Asia University, Taichung 413505, Taiwan

**Keywords:** dysphagia, older adults, prevalence, public health, assessment, community, nursing home, hospital

## Abstract

Background: This systematic review and meta-analysis aimed to estimate the pooled prevalence of dysphagia in older adults, subgrouping by recruitment settings and varying dysphagia assessment methods. Methods: Five major databases were systematically searched through January 2022. A random-effects model for meta-analysis was conducted to obtain the pooled prevalence. Results: Prevalence of dysphagia in the community-dwelling elderly screened by water swallow test was 12.14% (95% CI: 6.48% to 19.25%, I^2^ = 0%), which was significantly lower than the combined prevalence of 30.52% (95% CI: 21.75% to 40.07%, I^2^ = 68%) assessed by Standardized Swallowing Assessment (SSA) and volume-viscosity swallow test (V−VST). The dysphagia prevalence among elderly nursing home residents evaluated by SSA was 58.69% (95% CI: 47.71% to 69.25%, I^2^ = 0%) and by the Gugging Swallowing Screen test (GUSS) test was 53.60% (95% CI: 41.20% to 65.79%, I^2^ = 0%). The prevalence of dysphagia in hospitalized older adults screened by the 10-item Eating Assessment Tool was 24.10% (95% CI: 16.64% to 32.44%, I^2^ = 0%), which was significantly lower than those assessed by V-VST or GUSS tests of 47.18% (95% CI: 38.30% to 56.14%, I^2^ = 0%). Conclusions: Dysphagia is prevalent in the elderly, affecting approximately one in three community-dwelling elderly, almost half of the geriatric patients, and even more than half of elderly nursing home residents. The use of non-validated screening tools to report dysphagia underestimates its actual prevalence.

## 1. Introduction

Average life expectancy has risen steadily worldwide from 66.8 years in 2000 to 73.4 years in 2019 [1]. This number is expected to rise even higher by the year 2050, when one out of every six individuals on our planet would be 65 years old or older [2]. Oropharyngeal dysphagia, also known as deglutition disorders, is defined as the impairment of swallowing capacity, which is attributable to a variety of diseases and disorders [3]. As the term suggests, oropharyngeal refers to the oral and pharyngeal regions and is distinct from esophageal dysphagia. In this paper, we use the term dysphagia to mean oropharyngeal dysphagia. Dysphagia is a worrisome problem among the geriatric population [4]. Evidence indicated that advanced age might lead to a significant diminish in swallowing mechanisms even in the absence of underlying diseases [5,6,7,8]. A range of sensory-motor physiology of oropharyngeal function has been consistently proved to become progressively hyposensitive as people age [9,10,11,12]. The potential complications of dysphagia for older adults include undernutrition, dehydration, and especially aspiration pneumonia [13,14,15]. It significantly prolonged hospital length of stay [16] and negatively impacted patients’ quality of life [17]. Dysphagia was identified as a risk factor for mortality in nursing home residents [18], nearly half dysphagic nursing home occupants developed aspiration pneumonia in 12 months, with 45% mortality [19]. Given the negative consequences and poor outcomes, early and accurate recognition are essential to standardize diagnosis and improve future research [16]. There are a variety of screening tools and tests to identify dysphagia, including questionnaires [20,21], water swallow tests (WST) [22], multiple consistency tests such as the volume-viscosity swallow test (V-VST) or the Gugging Swallowing Screen (GUSS) [23,24], Standardized Swallowing Assessment (SSA) and so on [25,26]. Patients who fail the screening should be referred for further evaluation and treatment by dysphagia specialists [27]. Instrumental tools such as videofluoroscopy (VF) or fiberoptic endoscopic evaluation of swallowing (FEES) are sometimes needed to offer accurate measurement and select specific therapeutic strategies [28]. There has not been a universal consensus on an accurate procedure to identify dysphagia. Using different instruments might lead to inconsistency in reporting its prevalence. There has been a wide range of estimates of dysphagia prevalence in the elderly, with great disparities, ranging from 11.4% to 91.7% [29]. Up to date, there are knowledge gaps concerning the overall prevalence of dysphagia in older adults and how different dysphagia measurement methods influence the reported prevalence in this population. Therefore, this systematic review and meta-analysis aims to synthesize and analyze the totality of existing epidemiological studies on the prevalence of dysphagia in older adults to bridge these gaps. We hypothesized that the prevalence of dysphagia in older adults would be high, and the utilization of non-validated tools to report dysphagia could underestimate its actual prevalence.

## 2. Materials and Methods

This systematic review and meta-analysis followed the 2009 Preferred Reporting Items for Systematic Reviews and Meta-analyses (PRISMA) guidelines [30]. The searching process, assessment of the risk of bias and data extraction were independently conducted by two authors. In cases of discrepancies, the consensus was reached through group discussions with other authors.

### 2.1. Eligibility Criteria

Studies were eligible if they were observational studies published in English, which assessed the prevalence of dysphagia in older adults (≥60 years old) [31]. Studies were excluded if they contained neither prevalence nor sufficient original data to calculate the prevalence of dysphagia. We also excluded those studies that determined dysphagia by dichotomous question (yes, no), studies that assessed the prevalence of dysphagia in populations with specific diseases such as stroke, head injury, cardiopulmonary diseases, Parkinson’s disease, dementia, cancer, multiple sclerosis, myasthenia gravis, myositis, systemic sclerosis, and so forth. Case studies, reviews, editorials, or abstracts with no full text were also excluded in this investigation.

### 2.2. Search Strategy and Screening Process

A systematic literature search was conducted from inception through January 2022 of Pubmed, Excerpta Medica dataBASE (EMBASE), Web of Science, Cumulative Index of Nursing and Allied Health Literature (CINAHL), and Virtual Health Library Portal (VHL) libraries. The following search terms were primarily utilized: Dysphagia, Swallowing Disorders, Deglutition Disorders, Prevalence, Elderly, Older Adults, Older Individuals, Older People. The specific search terms for each library are detailed in Table S1. In addition, we have retrieved reference lists in relevant articles to gain more potential papers. Studies that were not available in full-text or missing data were requested directly by contacting the corresponding authors via email. Initially, duplicates were automatically removed by EndNote X9, and the apparent irrelevant studies were excluded through reviewing titles and abstracts. Two authors independently read full texts of potential articles to obtain studies that met the eligibility criteria. All hesitations were resolved by group discussion with other authors.

### 2.3. Assessment of the Risk of Bias

The Joanna Briggs Institute’s Critical Appraisal (JBIC) Checklist was utilized to estimate the methodological quality of the included studies. The JBIC Checklist encompasses nine items with four options: Yes, No, Unclear, and Not applicable. Studies scored of at least five are considered adequate quality [32].

### 2.4. Data Extraction

We extracted the required information and summarized in tables, for each study that met eligibility criteria, the study characteristics (first author’s name, year of publication, country), settings used to recruit participants, sample size, the average age of participants, gender ratio, tools to report dysphagia, and the prevalence of dysphagia.

### 2.5. Statistical Analysis

Statistical software RStudio Version 1.4.1717 (Integrated Development for R. RStudio, PBC, Boston, MA, USA) was used to analyze the data. It was expected that the heterogeneity among studies would be considerable. Thus, a random-effects model was chosen to obtain the pooled prevalence. We calculated the overall prevalence of dysphagia in the elderly. Subgroup analyses were conducted to investigate the sources of heterogeneity among studies. Hedges Q and I^2^ statistics were used to assess and quantify the magnitude of heterogeneity among studies. I^2^ values ranging between 25% and 50% were classified as low, between 50% and 75% as moderate, and 75% or above as high heterogeneity [33]. Statistical significance was set at *p* < 0.05.

## 3. Results

### 3.1. Study Selection

**Figure 1:**
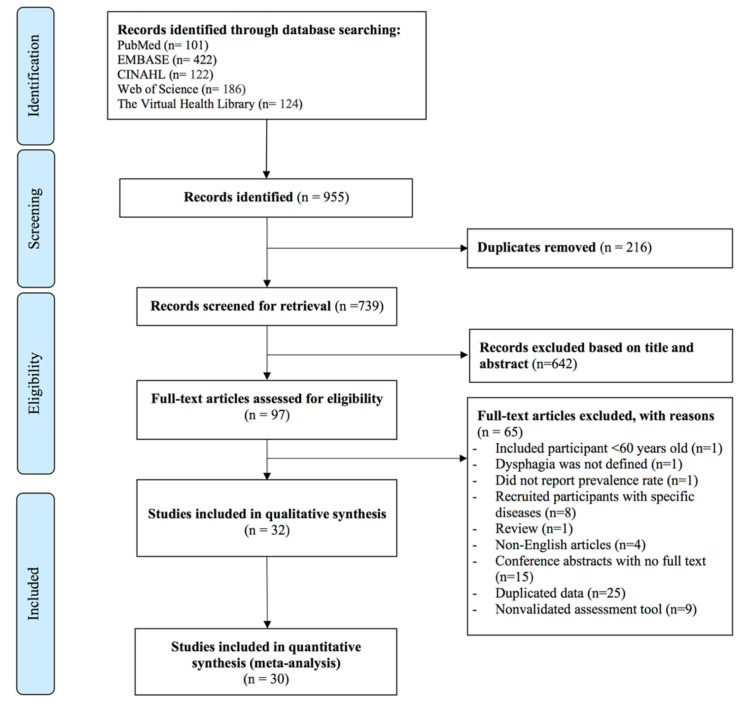
PRISMA diagram of the study selection process.

The systematic search strategy on five electronic libraries yielded a total of 955 abstracts: 101 from PubMed, 422 from Embase, 122 from CINAHL, 186 from Web of Science, and 124 from The Virtual Health Library. A manual search of the references cited in relevant studies yielded no additional articles. After removing 216 duplicates, of the 739 studies remaining, 642 studies were excluded by screening title and abstract, 97 full-texts were assessed for eligibility in accordance with the inclusion and exclusion criteria. Subsequently, 65 studies were excluded because of reasons as follows: 1 study included participants less than 60 years old, 1 study did not mention assessment tool for dysphagia, one study did not report prevalence rate, eight studies recruited participants with specific diseases, 1 review article, 4 non-English articles, 15 conference abstracts with no full text, 25 papers with duplicated data, and 9 studies used dichotomous question (yes, no) to determine dysphagia. Finally, a total of 32 studies were collected in this systematic review, of which 30 studies were synthesized in meta-analyses. The study selection process is shown in Figure 1.

### 3.2. Characteristics of Studies

Of the 32 included studies, 12 studies were from Europe and North America [34,35,36,37,38,39,40,41,42,43,44,45], 17 from Asia [46,47,48,49,50,51,52,53,54,55,56,57,58,59,60,61,62], 2 from Oceania [63,64], and 1 from South America [65]. Two studies recruited participants aged 80 years or older [34,41], reporting too high prevalences of dysphagia to be representative for the purpose of the present study. Therefore, we did not include these two studies in meta-analyses to avoid potentially misleading results. Of the 30 studies included in the meta-analysis, 11 studies recruited older adults in communities, 9 in nursing homes (also known as residential aged care facilities or aged care homes), and 10 in hospitals. There were different tools to report dysphagia in the included studies. These could be classified as four strategy criteria: questionnaires, water swallow tests, multiple consistency tests, and standardized swallowing assessments. Main characteristics of the included studies are shown in Table 1.

### 3.3. Study Methodology Evaluation

Study quality was assessed by the Joanna Briggs Institute’s Critical Appraisal Checklist, with the scores ranging from 6–9, suggesting that the methodology of the included studies was moderate to high. Study methodology evaluations are detailed in Table S2.

### 3.4. Prevalence of Dysphagia in the Elderly

Based on the random-effect model, the estimated overall prevalence of dysphagia in the elderly was 32.83% (95% CI: 26.63% to 39.35%, I^2^ = 99%, *p* < 0.001) (Figure S1). We conducted subgroup analyses based on different settings. The pooled estimates of dysphagia prevalence in older adults in community, nursing home, and hospital were 18.39% (95% CI: 15.28% to 21.71%, I^2^ = 95%), 46.98% (95% CI: 41.78% to 52.20%, I^2^ = 95%), and 37.98% (95% CI: 32.49% to 43.63%, I^2^ = 98%), respectively. Because of the high heterogeneity in meta-analyses of each setting, we conducted further subgroup analyses under the methods for measuring swallowing function.

#### 3.4.1. Prevalence of Dysphagia in Community-Dwelling Elderly

**Figure 2:**
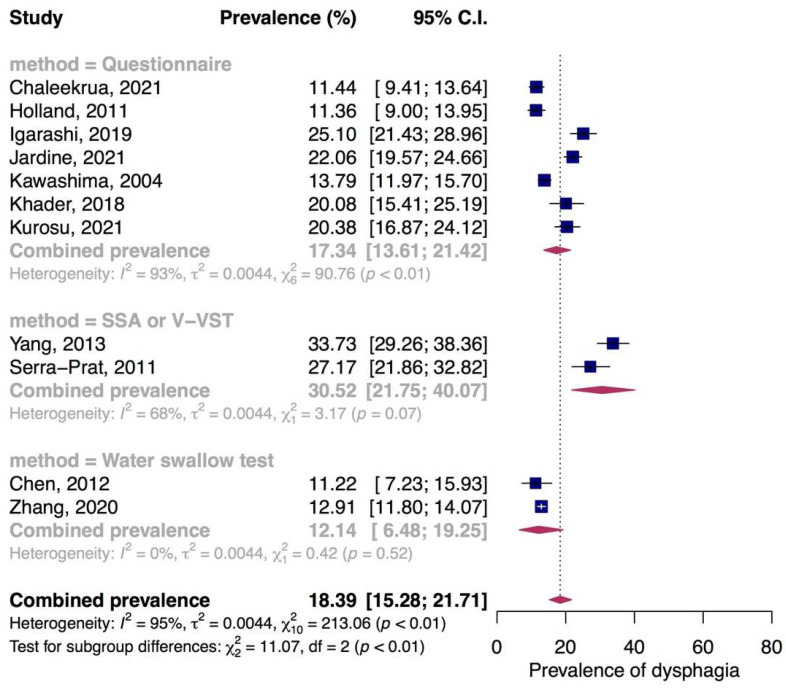
Prevalence of dysphagia in community-dwelling elderly. SSA, Standardized Swallowing Assessment; V-VST, volume-viscosity swallow test.

The highest prevalence (33.73%) of dysphagia in community-dwelling elderly was reported using Standardized Swallowing Assessment (SSA), followed by the prevalence of 27.17% reported by using V−VST. Due to the similarity in the description of these two assessments, we grouped the two studies in one subgroup with a combined prevalence of 30.52% (95% CI: 21.75% to 40.07%, I^2^ = 68%, *p* = 0.07). The estimated prevalence of 17.34% (95% CI: 13.61% to 21.42%, I^2^ = 93%, *p* < 0.01) was obtained by combining seven studies assessed dysphagia using questionnaires. Two studies using the water swallow test made up the prevalence of 12.14% (95% CI: 6.48% to 19.25%, I^2^ = 0%, *p* = 0.52) (Figure 2).

#### 3.4.2. Prevalence of Dysphagia in Nursing Home Residents

**Figure 3:**
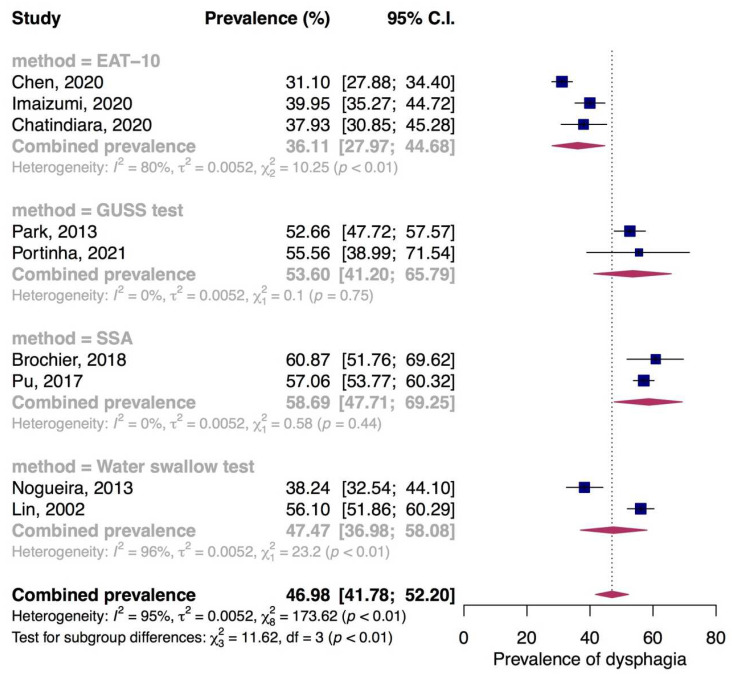
Prevalence of dysphagia in nursing home residents. EAT-10, 10-item Eating Assessment Tool; GUSS, Gugging Swallowing Screen test; SSA, Standardized Swallowing Assessment.

The highest prevalence of dysphagia in nursing home residents was shown in two studies performing SSA by qualified speech therapists with combined prevalence of 58.69% (95% CI: 47.71% to 69.25%, I^2^ = 0%, *p* = 0.44). Pooled prevalence of dysphagia based on the the Gugging Swallowing Screen (GUSS) test and water swallow test were 53.60% (95% CI: 41.20% to 65.79%, I^2^ = 0%, *p* = 0.75) and 47.47% (95% CI: 36.98% to 58.08%, I^2^ = 96%, *p* < 0.01), respectively. Combined prevalence from three studies screened dysphagia by the 10-item Eating Assessment Tool (EAT−10) was 36.11% (95% CI: 27.97% to 44.68%, I^2^ = 80%, *p* < 0.01) (Figure 3).

#### 3.4.3. Prevalence of Dysphagia in Hospitalized Older Adults

**Figure 4:**
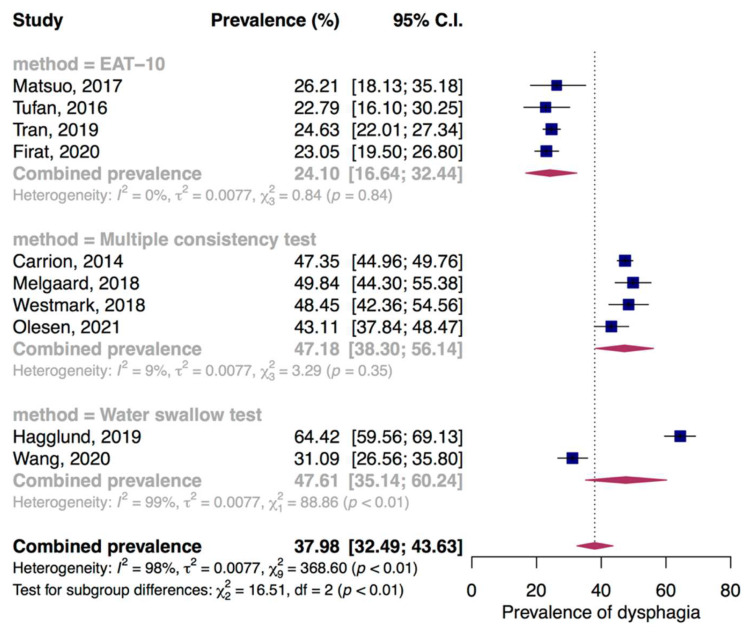
Prevalence of dysphagia in hospitalized older adults. EAT-10, 10-item Eating Assessment Tool; multiple consistency test, volume-viscosity swallow test or Gugging Swallowing Screen test.

There was the high consistency among four studies screened dysphagia in hospitalized older adults by EAT−10 with combined prevalence of 24.10% (95% CI: 16.64% to 32.44%, I^2^ = 0%, *p* = 0.84). While, the higher combined prevalence of 47.18% (95% CI: 38.30% to 56.14%, I^2^ = 0%, *p* = 0.71) was found when pooled four studies assessing multiple consistency tests (three studies examined dysphagia using V−VST and one study using GUSS test). Whereas, high discrepancy existed between two studies performing water swallow tests with pooled prevalence of 47.61% (95% CI: 35.14% to 60.24%, I^2^ = 99%, *p* < 0.01) (Figure 4).

## 4. Discussion

The purpose of this study is to systematically identify the totality of existing literature to estimate the prevalence of dysphagia in the geriatric population and to analyze the influence of different assessment methods on the reported prevalence. Of note, the accurate epidemiological data of dysphagia in the elderly is problematic to ascertain, which depends upon a variety of factors, including the recruited population in particular settings, different medical conditions, especially inconsistent methods and assessment procedures employed to define its presence in different epidemiological studies. Based on the included studies, the main findings of the current review were (1) the prevalence of dysphagia is high in older adults, and (2) the use of non-validated screening tools to report dysphagia underestimates its actual prevalence in older adults.

In clinical practice, a comprehensive swallowing assessment often includes a review of the patient’s socio-psychological and medical history, self-reported symptoms, cognitive-linguistic assessment, oral-peripheral examination, trials with different consistencies of food and liquids or a review of usual eating patterns, and intervention trials [27,66]. While SSA approximates the comprehensive swallowing assessment, especially when performed by speech-language pathologists [50,57,67]; multiple consistency tests (V-VST or GUSS) are a constituent of the comprehensive swallowing assessment. The V-VST was designed to investigate the clinical impairment in the safety and efficiency of swallowing three different viscosities (water, nectar, and pudding) with three different volumes (5, 10, and 20 mL) [23]. The GUSS test has two sections, including indirect swallowing trials and direct swallowing trials with three different consistencies (solid, semisolid, and liquid) [24]. Either the GUSS test with 0.955 sensitivity and 0.944 specificity [68] or the V-VST with 0.94 sensitivity and 0.88 specificity [69] is ideally suited to identify dysphagia among the older population. Furthermore, healthcare practitioners who perform dysphagia identification should get specialized training in this field. Some questionnaires were used to identify the prevalence of dysphagia, which has not previously been validated among the aging population. The investigation of swallowing dysfunctions should not be confined to self-reported measures; particularly, senior citizens with cognitive and sensory impairments may not be aware of their swallowing abnormalities [70]. Despite the fact that there has not been a validation study of EAT-10 in the detection of dysphagia in older adults, this questionnaire was the most frequently used in the included studies; its accuracy has been assessed in a population with 10.8% neurodegenerative diseases, 55% stroke, and 34.2% elderly, with the sensitivity and specificity of 0.82 and 0.85, respectively [69]. In low-resource communities or remote areas, EAT-10 could be used to identify individuals who may require a more thorough assessment. Water swallow tests were validated among individuals with various etiologies encompassing a small group of older individuals with a sensitivity of 85.5% and a specificity of 50% [71]. These tests do not consider the safety and efficacy of swallowing different consistencies. Furthermore, a lack of consensus on the test quantity and endpoints in WST resulted in heterogeneity among studies.

Regardless of subgroups classified by different recruitment settings, the prevalence of dysphagia among the older population estimated by using non-validated methods was significantly lower than those assessed by validated methods. When considering subgroup analyses with SSA, V-VST, or GUSS as the assessment method only, up to 30% of community-dwelling elderly have dysphagia, it presents in almost half of hospitalized elderly and more than half of nursing home residents. In people aged 80 and over, the prevalence of dysphagia has been found to be even much higher [34,41]. Nevertheless, in a survey of 150 health professionals across 29 countries working in acute hospitals, rehabilitation facilities, or the community, 93 out of 150 (62 percent) reported that their health services do not or solely occasionally screen the elderly for dysphagia [72]. More attention should be paid to swallowing problems in older adults as screening its presence potentially ameliorates morbidity and mortality [72,73].

There appears to be a scarcity of studies investigating the prevalence of dysphagia in the elderly by instrumental assessments such as VF or FEES. Studies should be further developed to bridge this gap. Further comparative studies pertaining to the assessment methods of dysphagia are needed to identify whether these approaches affect the secondary consequences of dysphagia. Much remains to be done to establish an international consensus on screening dysphagia in the elderly in community as well as early detection and management of dysphagia in residential care and clinical settings.

We acknowledge several potential limitations of this systemic review and meta-analysis. Firstly, we may have missed relevant studies that were (1) not published; (2) abstracts without full text; (3) published in other languages than English. Secondly, there is significant heterogeneity, even after subgroup analyses, due to multifactorial nature of dysphagia assessment including differences in (1) assessments; (2) recruitment settings; (3) health conditions; (4) assessors. Due to this limitation, only subgroups were analyzed. Finally, dysphagia can be caused by a variety of diseases and disorders, such as stroke, Parkinson’s disease or dementia, and so on, which are common among the elderly. In this review, in addition to the exclusion of studies assessing dysphagia in specific medical conditions, several collected studies also excluded individuals with neurological diseases or other medical conditions in their investigations, which might underestimate the true prevalence of dysphagia in the older population.

## 5. Conclusions

In conclusion, dysphagia is prevalent in older adults. It affects around 30% of community-dwelling elderly, almost 50% of geriatric patients, and above 50% of nursing home residents. The use of non-validated screening tools to report dysphagia underestimates its actual prevalence. Given the high prevalence and associated poor outcomes, there is a need to better standardize how studies assess and report dysphagia. This is needed to advance research in this highly important field as well as potentially improve patient outcomes.

## Figures and Tables

**Table 1:** Characteristics of included studies (*n* = 32).

No.	First Author, Year	Country/Region	Sample Size	Age and Mean Age (Years)	%Women	Specific Eligibility	Tool to Report Dysphagia	Strategy Criteria	Dysphagia Prevalence
**Community (*n*** ** = ** **12)**									
**1**	Chaleekrua, 2021 [46]	Thailand	874	≥60 (69.70 ± 6.79)	66.2	Excluded: individuals with neurological diseases, head and neck cancer	EAT-10	Questionnaire	11.4%
**2**	Chen, 2012 [47]	Taiwan	216	≥65 (74.2 ± 6.33)	72.2	Excluded: neurological diseases, stroke < 3 months, cancer, chronic pulmonary diseases	90 mL WST	Water swallow test	11.2%
**3**	Fernandez, 2014 [34]	USA	47	85 to 94 (89 ± 2.9)	100		90 mL WST	Water swallow test	72%
**4**	Holland, 2011 [35]	UK	634	69–98 (81 ± 5)	76.5	Excluded: individuals with history of neurological diseases	Sydney Swallow Questionnaire	Questionnaire	11.4%
**5**	Igarashi, 2019 [48]	Japan	510	≥65 (75.0 ± 7.2)		Independent older people	EAT-10	Questionnaire	25.1%
**6**	Jardine, 2021 [63]	New Zealand	1020	≥65 (75.22 ± 6.24)	61		EAT-10	Questionnaire	22.1%
**7**	Kawashima, 2004 [49]	Japan	1313	≥65 (74.1 ± 6.9)	56.2		Dysphagia Screening Questionnaire	Questionnaire	13.8%
**8**	Yang, 2013 [50]	South Korea	415	≥65 74 (65–95)	53.1		Standardized Swallowing Assessment	Standardized Swallowing Assessment	33.7%
**9**	Zhang, 2020 [51]	China	3361	≥65 (72.64 ± 6.10)	51.8	Excluded: bed-ridden, hearing disorders, serious physiological or psychological illness	30 mL WST	Water swallow test	12.9%
**10**	Khader, 2018 [52]	India	259	≥60 (66.2 ± 6.2)	32.8	Excluded: patients with aspiration risk, neurological diseases, upper GI malignancy, oropharyngeal diseases	Swallowing Disturbance Questionnaire	Questionnaire	20.1%
**11**	Serra-Prat, 2011 [36]	Spain	254	≥70 (78.2 ± 5.6)	46.5	Excluded: persons with a life expectancy of less than 3 months	V-VST	Multiple consistency test	27.2%
**12**	Kurosu, 2021 [61]	Japan	476	≥65 (78.5 ± 11.12)	64.4		EAT-10	Questionnaire	20.4%
**Nursing homes (*n*** ** = ** **9)**									
**13**	Brochier, 2018 [65]	Brazil	115	≥60	67		Standardized Swallowing Assessment	Standardized Swallowing Assessment	60.9%
**14**	Chen, 2020 [53]	China	775	≥60 (81.3 ± 9.3)	60.6	Excluded: intellectual disabilities	EAT-10	Questionnaire	31.1%
**15**	Nogueira, 2013 [37]	Portugal	272	≥65 (82 ± 10)	75	Excluded: low level of consciousness	90 mL WST	Water swallow test	38.2%
**16**	Park, 2013 [54]	South Korea	395	≥65 (80.7 ± 8.0)	76.5		GUSS	Multiple consistency test	52.7%
**17**	Lin, 2002 [55]	Taiwan	611	≥65 (77 ± 10.8)	48.1		90-mL timed swallowing test	Water swallow test	56.1%
**18**	Imaizumi, 2020 [56]	Japan	413	≥65 (84.4)	73.1		EAT-10	Questionnaire	40%
**19**	Pu, 2017 [57]	Hong Kong	878	≥60 (84.5 ± 7.9)	70.1	Excluded: not cognizant enough or unfit to be assessed on the day of examination	Standardized Swallowing Assessment	Standardized Swallowing Assessment	57.1%
**20**	Portinha, 2021 [38]	Portugal	36	≥65 (88.0 ± 5.6)	63.8		GUSS	Multiple consistency test	55.6%
**21**	Chatindiara, 2020 [64]	New Zealand	174	≥65 (85.5 ± 7.5)		Excluded: palliative care, dementia, previoulsy diagnosis with swallow disorders, cancer of the larynx or psychiatric eating disorders	EAT-10	Questionnaire	37.9%
**Hospital (*n*** ** = ** **11)**									
**22**	Carrion, 2014 [40]	Spain	1662	≥70 (85.1 ± 6.23)	61.7	Excluded: terminal patients	V-VST	Multiple consistency test	47.4%
**23**	Mateos, 2020 [41]	Spain	329	≥80 (93.5 ± 4.1)	68.4	Excluded: end-of life situations, low level of consciousness, enteral nutrition	V-VST	Multiple consistency test	82.4%
**24**	Matsuo, 2017 [60]	Japan	103	≥65 (80.5 ± 7.9)	56.3	Excluded: neurological diseases, head and neck cancer	EAT-10	Questionnaire	26.2%
**25**	Westmark, 2018 [42]	Denmark	258	≥60 (83.1 ± 7.7)	54	Excluded: delirium, severe dementia, terminal patients	V-VST	Multiple consistency test	48.5%
**26**	Tufan, 2016 [62]	Turkey	136	≥65 (74.6 ± 6.6)	54.4		EAT-10	Questionnaire	23%
**27**	Wang, 2020 [58]	China	386	≥65 (74.76 ± 6.19)	51.6	Excluded: Parkinson’s disease, stroke, dementia, myasthenia gravis, head and neck cancer, gastric tubes, tracheal intubation, diagnosed swallowing dysfunction, disorder of consciousness	30 mL WST	Water swallow test	31.1%
**28**	Hagglund, 2019 [39]	Sweden	385	≥65 81 (65–98)	53.4	Excluded: People with end-of-life care, insufficient cognitive capacity	150-mL timed swallowing test	Water swallow test	64%
**29**	Tran, 2019 [59]	Vietnam	1007	≥65 (75.5)	58.3	Excluded: patients with mute, deaf, psychosis, on ventilator, coma, dementia, trauma	EAT-10	Questionnaire	24.6%
**30**	Melgaard, 2018 [43]	Denmark	313	≥60 (83.1 ± 7.8)	56	Excluded: terminal illness, dementia, delirium, hospitalized for less than 24 h	V-VST	Multiple consistency test	50%
**31**	Fırat, 2020 [44]	Turkey	512	≥60 72.1 ± 7.3		Excluded: severe dementia, head and neck cancer, or acute diseases with clinical deterioration	EAT-10	Questionnaire	23%
**32**	Olesen, 2021 [45]	Denmark	334	≥65 78.5 (73–85)	47.7		WST + GUSS	Multiple consistency test	43.1%

*n*, number of studies; EAT-10, 10-item Eating Assessment Tool; WST, water swallow test; V-VST, volume-viscosity swallow test; GUSS, Gugging Swallowing Screen test.

## Data Availability

The data that support the findings of this study are available from the corresponding author, upon reasonable request.

## Supplementary Materials

The following supporting information can be downloaded at: https://www.mdpi.com/article/10.3390/jcm11092605/s1, Figure S1: Pooled prevalence of dysphagia in the elderly of all included studies; Table S1: Search Strategy; Table S2: Study Quality Assessment.
