# Supplementary material for: Prevalence and Methods for Assessment of Oropharyngeal Dysphagia in Older Adults: A Systematic Review and Meta-Analysis
Source: J Clin Med. 2022 May 6;11(9):2605. doi: 10.3390/jcm11092605 (PMC9104951; doi:10.3390/jcm11092605)
Supplement: Supplementary file 1 [file jcm-11-02605-s001.zip › jcm-1697382-supplementary.pdf]

Prevalence and Methods for Assessment of Oropharyngeal Dysphagia in Older Adults:

A Systematic Review and Meta-Analysis

Supplementary Figure and Table

Study	Cases	Samplesize	Prevalence	95% C.I
Brochier, 2018	70	115	60.87	[51.33; 69.84]
Chen, 2020	241	775	31.10	[27.85; 34.49]
Nogueira, 2013	104	272	38.24	[32.43; 44.30]
Park, 2013	208	395	52.66	[47.60; 57.67]
Lin, 2002	299	533	56.10	[51.77; 60.36]
Hagglund, 2019	248	385	64.42	[59.41; 69.20]
Carrion, 2014	787	1662	47.35	[44.93; 49.79]
Matsuo, 2017	27	103	26.21	[18.04; 35.80]
Tufan, 2016	31	136	22.79	[16.04; 30.77]
Wang, 2020	120	386	31.09	[26.50; 35.97]
Tran, 2019	248	1007	24.63	[21.99; 27.41]
Olesen, 2021	144	334	43.11	[37.73; 48.62]
Melgaard, 2018	156	313	49.84	[44.17; 55.52]
Pu, 2017	501	878	57.06	[53.71; 60.36]
Westmark, 2018	125	258	48.45	[42.21; 54.73]
Imaizumi, 2020	165	413	39.95	[35.19; 44.85]
Firat, 2020	118	512	23.05	[19.47; 26.94]
Chatindiara, 2020	66	174	37.93	[30.70; 45.58]
Portinha, 2021	20	36	55.56	[38.10; 72.06]
Chaleekrua, 2021	100	874	11.44	[9.41; 13.74]
Chen, 2012	23	205	11.22	[7.25; 16.36]
Holland, 2011	72	634	11.36	[8.99; 14.09]
Igarashi, 2019	128	510	25.10	[21.39; 29.10]
Jardine, 2021	225	1020	22.06	[19.55; 24.73]
Kawashima, 2004	181	1313	13.79	[11.97; 15.77]
Yang, 2013	140	415	33.73	[29.20; 38.51]
Zhang, 2020	434	3361	12.91	[11.80; 14.09]
Khader, 2018	52	259	20.08	[15.37; 25.48]
Serra−Prat, 2011	69	254	27.17	[21.79; 33.08]
Kurosu, 2021	97	476	20.38	[16.85; 24.28]
Random effects model		.	32.83	[26.63; 39.35]

Heterogeneity: I^2 = 99%, τ^2 = 0.0348, χ^2_{29} = 2362.70 (p = 0)

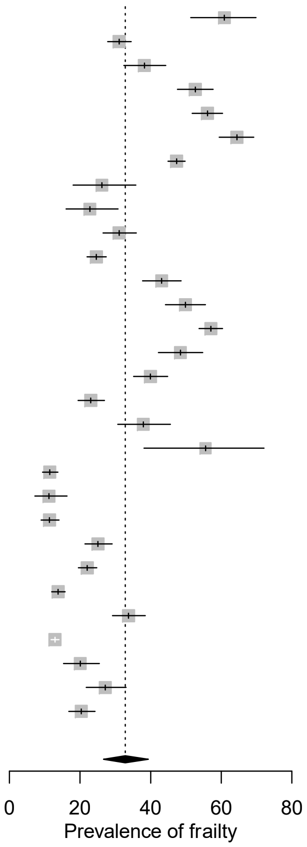

Figure S1. Overall prevalence of dysphagia in the elderly.

Table S1. Search strategy.

Database	Search Strategy
**EMBASE**	(dysphagia:ab,ti OR 'swallowing disorders':ab,ti OR 'deglutition disorders':ab,ti) AND prevalence:ab,ti AND (aged:ab,ti OR elders:ab,ti OR 'older adults':ab,ti OR 'older people':ab,ti OR 'older individuals':ab,ti OR 'nursing home':ab,ti OR 'institutionalised elderly':ab,ti OR 'institutionalized older people':ab,ti OR geriatric:ab,ti)
**CINAHL**	(Dysphagia OR Esophageal Dysphagia OR Oropharyngeal Dysphagia OR Swallowing Disorders OR Deglutition Disorders) AND Prevalence AND (Elderly OR Frail Elders OR Older Adults OR older people OR older individuals OR nursing home OR institutionalized elderly OR Institutionalized Older People OR Geriatric)
**Web of Science**	(((((AB=(Elderly)) OR AB=(Older people)) OR AB=(nursing home)) OR AB=(institutionalized elderly)) OR AB=(older adults)) AND AB=(prevalence) AND (((AB=(Dysphagia)) OR AB=(Swallowing Disorders)) OR AB=(Deglutition Disorders))
**Pubmed**	(Dysphagia[Title/Abstract] OR Esophageal Dysphagia[Title/Abstract] OR Oropharyngeal Dysphagia[Title/Abstract] OR Swallowing Disorders[Title/Abstract] OR Deglutition Disorders[Title/Abstract]) AND Prevalence[MeSH Terms] AND (Elderly[Title/Abstract] OR Frail Elders[Title/Abstract] OR Older Adults[Title/Abstract] OR older people[Title/Abstract] OR older individuals[Title/Abstract] OR nursing home[Title/Abstract] OR institutionalized elderly[Title/Abstract] OR Institutionalized Older People[Title/Abstract] OR Geriatric[Title/Abstract])
**The Virtual Health Library (VHL)**	((ti:(Dysphagia)) OR (ti:(Swallowing Disorders)) OR (ti:(Deglutition Disorders))) AND ((ab:(prevalence)) OR (ti:(epidemiology)) OR (ti:(cross-sectional)) OR (ti:(rate))) AND ((ab:(elderly)) OR (ti:(Older Adults)) OR (ti:(nursing home)) OR (ti:(Older)) OR (ti:(Geriatric)))

Table S2. Study Quality Assessment.

Author	Was the sample frame appropriate to address the target population?	Were study participants sampled in an appropriate way?	Was the sample size adequate?	Were the study subjects and the setting described in detail?	Was the data analysis conducted with sufficient coverage of the identified sample?	Were valid methods used for the identification of the condition?	Was the condition measured in a standard, reliable way for all participants?	Was there appropriate statistical analysis?	Was the response rate adequate, and if not, was the low response rate managed appropriately?	Total number of "Yes"
Chaleekrua,2021	Y	Y	Y	Y	Y	Y	Y	Y	Y	9
Chen, 2012	Y	N	N	N	Y	Y	Y	Y	Y	6
Fernandez, 2014	N	N	N	Y	N	Y	Y	Y	Y	5
Holland, 2011	Y	Y	N	Y	N	Y	Y	Y	Y	7
Igarashi, 2019	Y	Y	Y	Y	Y	Y	Y	Y	Y	9
Jardine, 2021	Y	Y	Y	Y	Y	Y	Y	Y	Y	9
Kawashim,2004	Y	Y	Y	Y	Y	Y	Y	Y	Y	9
Yang, 2013	Y	Y	Y	Y	Y	Y	Y	Y	Y	9
Zhang, 2020	Y	N	N	Y	Y	Y	Y	Y	Y	7
Khader, 2018	Y	Y	N	Y	Y	Y	Y	Y	Y	8
Serra-Prat, 2011	Y	Y	Y	Y	Y	Y	Y	Y	Y	9
Kurosu, 2021	Y	Y	Y	Y	Y	Y	Y	Y	Y	9
Brochier, 2018	Y	N	N	Y	Y	Y	Y	Y	Y	7
Chen, 2020	Y	Y	Y	Y	Y	Y	Y	Y	Y	9
Nogueira, 2013	Y	Y	N	Y	Y	Y	Y	Y	Y	8
Park, 2013	Y	Y	Y	Y	Y	Y	Y	Y	Y	9
Lin, 2002	Y	Y	Y	Y	Y	Y	Y	Y	Y	9
Dai, 2017	Y	Y	Y	Y	Y	Y	Y	Y	Y	9
Susana, 2021	N	N	N	N	Y	Y	Y	Y	Y	5
Idah, 2020	Y	Y	N	Y	Y	Y	Y	Y	Y	8
Imaizumi, 2020	Y	Y	Y	Y	Y	Y	Y	Y	Y	9
Hagglund, 2018	Y	Y	N	Y	Y	Y	Y	Y	N	7
Carrion, 2014	Y	Y	Y	Y	Y	Y	Y	Y	Y	9
Jesus, 2020	N	N	N	Y	Y	Y	Y	Y	Y	6

Matsuo, 2017	Y	Y	Y	Y	Y	Y	Y	Y	Y	9
Westmark, 2018	Y	Y	Y	Y	Y	Y	Y	Y	Y	9
Tufan, 2016	Y	Y	N	Y	Y	Y	Y	Y	Y	8
Wang, 2020	Y	Y	Y	Y	Y	Y	Y	Y	Y	9
Tran, 2019	Y	Y	Y	Y	Y	Y	Y	Y	Y	9
Melgaard, 2018	Y	Y	N	Y	Y	Y	Y	Y	Y	8
Firuzan, 2020	Y	Y	Y	Y	Y	Y	Y	Y	Y	9
Olesen, 2021	Y	Y	Y	Y	Y	Y	Y	Y	N	8
